# Self-assembled miR-134-5p inhibitor nanoparticles ameliorate experimental bronchopulmonary dysplasia (BPD) via suppressing ferroptosis

**DOI:** 10.1007/s00604-023-06069-3

**Published:** 2023-11-30

**Authors:** Jiang Lan, Xu Chen, Fengdan Xu, Fangfei Tao, Liyuan Liu, Rui Cheng, Ning Li, Ya Pan

**Affiliations:** 1 Shenzhen Longhua Maternity and Child Health Care Hospital, Shenzhen, 518000 China; 2 Hongqiao International Institute of Medicine, Tongren Hospital, Shanghai Jiao Tong University School of Medicine, Shanghai, 200336 China; 3 Children’s Hospital Affiliated to Nanjing Medical University (Nanjing Children’s Hospital), Nanjing, 210008 China; 4 Dongguan Children’s Hospital Affiliated to Guangdong Medical University, Dongguan, 523325 China

**Keywords:** Experimental bronchopulmonary dysplasia, Ferroptosis, Luminescence imaging, Dynamic light scattering, TEM, Nanometer materials, miR-134-5p, Nanoparticles

## Abstract

Bronchopulmonary dysplasia (BPD) is a chronic lung disease in premature infants with increased levels of reactive oxygen species (ROS) and ferroptosis. Herein, we designed a peptide-based nanoparticle to deliver therapeutic molecules to pulmonary, thereby ameliorating BPD. The BPD-induced damages of lung tissues were detected by H&E and immunohistochemistry staining. Inflammatory cytokines, Fe^2+^, and ROS levels were quantified by the indicated kits, respectively. The targeting relationship was verified by luciferase reporter assay and pull-down assay. Subsequently, self-assembled miR-134-5p inhibitor nanoparticles with pulmonary epithelial cell-targeting were synthesized. The characteristics were detected by transmission electron microscopy, luminescence imaging, and dynamic light scattering. A significant ferroptosis was observed in the BPD mice. The protein level of GPX4 was decreased significantly compared to the control group. Constantly, miR-134-5p showed positive regulation on ferroptosis by targeting GPX4. The designed nanoparticles were mainly accumulated in the lung region. Besides, it ameliorated experimental bronchopulmonary dysplasia via suppressing ferroptosis, in vivo and in vitro. Our findings provided a miR-134-5p/GPX4 axis in regulating ferroptosis of BPD and prompted the potential of applying the peptide-based nanoparticle to BPD treatment.

**Supplementary Information:**

The online version contains supplementary material available at 10.1007/s00604-023-06069-3.

## Introduction

Bronchopulmonary dysplasia (BPD) is a severe lung disease with immature lung development and occurs in almost half of extremely preterm infants [1]. Reduced alveolar numbers, enlarged alveolar volume, and associated vascular disorders are significant characterizations of BPD. In addition to impairment of lung functions, these pathological factors may lead to long-term physical and intellectual developmental disorders and can be fatal [2]. One fundamental cause of preterm infant BPD is the accumulation of oxidative stress [3].

ROS are highly reactive chemicals that oxidize intracellular compounds, resulting in inflammation and cell death [4]. Glucocorticoids and antioxidants have ameliorated BPD by reducing inflammation and eliminating ROS [5]. However, the primary concerns in clinics are the adverse effects of overdosing on glucocorticoids and the relatively short half-life of antioxidants. Thus, an alternative strategy is urgently needed.

Ferroptosis is an iron-dependent programmed cell death initiated by oxidative damage. Unlike other cell death patterns, ferroptosis is mainly characterized by excessive lipid peroxide accumulation, mitochondria shrinkage, and increased mitochondrial membrane density [6]. The acknowledged ferroptosis-related genes include nuclear factor erythroid 2-related factor 2 (NRF2), acyl-CoA synthetase long-chain family member 4 (ACSL4), and mitochondrially encoded cytochrome c oxidase II (COX2). It also results in the reduction of the core enzyme glutathione peroxidase 4 (GPX4), which regulates the antioxidant system [glutathione (GSH) system] [7]. GPX4 is an intracellular enzyme that depletes excessive ROS, thereby serving as an inhibitor for ferroptosis. In recent years, multiple studies have showed that ferroptosis induced by dysregulation of GPX4 is closely associated with various refractory diseases, such as neurodegenerative diseases [8], acute kidney injury [9], and several malignant tumors [10]. However, the interplay between ferroptosis and BPD has not been well studied yet.

MicroRNAs (miRNAs) are small non-coding RNAs, which negatively regulate gene expression by binding to the untranslated region of target gene mRNA (3′-UTR) to mediate the post-transcriptional silencing of the target genes [11]. More than 1000 human-related miRNAs have been identified, which are widely involved in cell proliferation, differentiation, and apoptosis [12]. Recent studies have found that miRNAs can be potential therapies and therapeutic targets in many diseases and have received widespread attention. In recent years, overwhelming studies have reported that miRNAs play a critical role in the pathophysiology of BPD [13–15]. Based on a previous miRNA profiling in the BPD C57BL/6 mouse model, we noticed that miR-134-5p is significantly upregulated in the lung tissues of BPD mice and is highly conserved between humans and mice [16]. However, the role of miR-134-5p needed to be elucidated further in BPD.

The vital step of miRNA-based gene therapy depends on the effective delivery of nucleic acids to avoid the degradation of miRNAs or miRNA inhibitors in an environment of nuclease-rich body fluids. Meanwhile, ensuring the miRNAs or the inhibitors are accurately delivered to target cells is essential for avoiding harmful side effects. Therefore, it is vital to select a suitable carrier. Presently, viral and non-viral vectors are nucleic acid vectors used clinically [17]. Advancements in nanotechnology have led to newer nano-composites such as non-viral nucleic carriers [18]. Due to the surface effect, small size effect and macroscopic quantum tunneling effect significantly differ in functions and properties from micron-level materials of the same composition. Furthermore, significant progress has been achieved in translating the application of self-assembled nanomaterials to clinics [19]. Compared with traditional gene carriers, there is a more excellent choice of nanocarriers that can be customized according to actual needs. The advantages of nanomaterials include good systemic circulation stability, high transfer efficiency, and biosafety in vivo and in vitro. Hence, excellent application prospects are attached to the self-assembled nanomaterials.

In this work, we aimed to verify the existence of ferroptosis in BPD mice and investigate the underlying relationship between miR-134-5p and hyperoxia-triggered ferroptosis. We then designed a targeted ROS-responsive nanocarrier (PCC-R8-ROS @miR-134-5p inhibitor) to deliver miR-134-5p inhibitor to the alveolar epithelial cells for BPD theranostic applications, providing an alternative strategy for BPD treatment.

## Materials and methods

### Construction and characterization of nanocarrier PCC-R8-ROS

The R8 was constructed via standard solid phase peptide synthesis (SPPS) procedure, followed by the attaching of clenbuterol. The ROS-responsive pro-fluorophore was then conjugated to the short peptide via Cu(I)-catalyzed azide-alkyne cycloaddition (CuAAC). High-performance liquid chromatography (HPLC), MALDI-TOF–MS, and UV–vis spectra confirmed the successful conjugation. The ROS responsiveness of PCC-R8-ROS was tested by recording the fluorescence spectra before and after incubating with H_2_O_2_ (AAPR555-A,pythonbio,China) with the nanocarrier.

### Preparation and characterization of PCC-R8-ROS@ miR-134-5p inhibitor

The synthetic nanocarrier (PCC-R8-ROS) was mixed with miR-134-5p inhibitors at different molar ratios in PBS buffer, and the mixture was allowed to stand at room temperature for 30 min. The encapsulation of miR-134-5p was analyzed by 4% agarose gel page. After preparation, PCC-R8-ROS @miR-134-5p were dipped into a carbon-coated copper grid. After 10 min, excess solution was carefully removed; subsequently, it was visualized using TEM (transmission electron microscopy). The hydrodynamic sizes of PCC-R8-ROS @miR-134-5p were measured by a DLS instrument.

### Mice model of BPD

All animal experiments were performed strictly according to the guidelines for using laboratory animals of the Institutional Animal Care and Use Committee of Shanghai Tongren Hospital. Pregnant BALB/c mice with similar gestation periods were purchased from Kaixue Biotech Co., Ltd. The BPD mouse model was established according to the previous study with a slight modification [20]. The mice were divided into the control (Ctrl) and BPD group to investigate the hyperoxia-induced damage on lung development. For investigating the therapeutic effects of self-assembled miR-134-5p, the mice were divided into Ctrl, BPD, miR-134-5p inhibitor (miR-134-5p Knockdown, miR-134-5p KD), PCC-R8-ROS @miR-NC, R8-ROS @miR-134-5p inhibitor (R8-ROS @miR-134-5p KD), and PCC-R8-ROS @miR-134-5p inhibitor (PCC-R8-ROS @miR-134-5p KD) group. Five mice were assigned to each group. Within 24 h of birth, mice in control groups were lived in room air (21% O_2_), while mice in the other groups were exposed to 85% O_2_ for 7 days to develop BPD-like symptoms. After that, the BPD mice recovered at room air until postnatal day 14. The nursing conditions for Ctrl and BPD mice were the same (50–60% humidity, 25 ± 2 °C). After modeling, the mice were euthanized, and the lung, heart, liver, and kidney tissues were resected and immediately frozen in liquid nitrogen for further investigation.

CMV-mmu-miR-134-5p inhibitors (1 × 10^5^ pfu/mL, 50 μL) were intravenous injected into the tail the mice in the miR-134-5p inhibitor group at postnatal day 0. The dosage of PCC-R8-ROS in mice was equivalent to PCC-R8-ROS @miR-134-5p inhibitor, calibrated by CMV‐mmu‐miR‐134-5p inhibitors (1 × 10^5^ pfu/mL, 50 μL).

### H&E staining

The resected tissues were embedded, sliced, and rehydrated before staining. Hematoxylin was first used to stain the sections for 10 min, followed by eosin for 2 min. After the routine dehydration process, the sections were sealed using a cover slip and observed under an optical microscope (NIKON ECLIPSE E100).

### Immunohistochemistry (IHC)

The deposition of pulmonary-associated surfactant protein (SFTPC; SP-C) in the lung tissues was visualized by IHC assay. The lung tissues were fixed, embedded, and then sliced into 5-μm sections. These sections were incubated with 3% H_2_O_2_ which dissolved in methanol (M871889,Macklin,China) for 10 min in order to blockade endogenous peroxidase. Next, the sections were incubated with goat serum (abs933; Absin Biotech Co., Ltd.) for another 20 min at room temperature and then incubated with anti-SFTPC (1:1000, ab211326, Abcam) primary antibody at 4 ℃ overnight. This primary antibody staining was followed by incubation with goat anti-mouse IgG H&L (HRP) (ab205719; Abcam) secondary antibody. Metal-Enhanced DAB Substrate Kit (DA1016; Solarbio Science & Technology Co., Ltd.) and hematoxylin were used to develop the sections.

### TUNEL assay for the lung sections

TUNEL staining assay was performed using the TUNEL Cell Apoptosis Detection Assay kit (G1501; Servicebio Technology Co., Ltd.). Firstly, the sections were deparaffinized and rehydrated; next were digested by Proteinase K for 25 min at 37 °C. Add permeabilize working solution to cover objective tissue, then incubate at room temperature for 20 min. After equilibrium at room temperature, take appropriate amount of TDT enzyme, dUTP, and buffer in the TUNEL kit according to the number of slices and tissue size and mix at 1:5:50 ratio, incubate at 37 ℃ for 2 h. DAPI (G1012, Servicebio) counterstain in nucleus, then coverslip with anti-fade mounting medium, microscopic examination, and collecting images through fluorescence microscope. Nucleus is blue by labeling with DAPI. TUNEL assay kit is labeled with FITC. Positive apoptosis cells are green.

### Cell culture

Mouse lung epithelial (MLE-12) cells were purchased from the Cell Bank of the Typical Culture Collection Committee of the Chinese Academy of Sciences and were cultured in Dulbecco’s modified Eagle medium (DMEM) containing 10% fetal bovine serum (FBS) and 100 mL streptomycin/penicillin at 37 ℃ with 5% CO_2_ for further experimental analysis. Hyperoxia was created with 95% O_2_/5% CO_2_ conditions (*v*/*v*) at 37 ℃.

### Cell transfection

293T cells were purchased from Procell Biotech Co., Ltd. and cultured in completed DMEM (with 10% FBS and 1% P/S). The miR-134-5p inhibitor or mimic (Biomics Biotech Co., Ltd.) were transfected into 2.5 × 10^6^ MLE-12 cells using Lipofectamine 2000 (Invitrogen) according to the manual. The subsequent experiments were conducted 24 h after transfection. The sequences used were: miR-134-5p inhibitor: 5′-ACACUGACCAACUGGUCUCCCC-3′ and miR-134-5p mimic: 5′-UGUGAC UGGUUGA CC AGAGGGG-3′.

### Cellular uptake of FAM-labeled PCC-R8-ROS-miR-134-5p inhibitor

293T cells or MLE-12 cells were grown in six-well tissue culture plates on glass coverslips before the experiment. FAM-labeled PCC-R8-ROS@miR-134-5p inhibitor was added to the cell culture system for co-incubation. After 24 h, the cellular uptake efficacy of FAM-labeled PCC-R8-ROS-miR-134-5p inhibitor was evaluated by confocal microscopy.

### Ferroptosis-related markers and inflammatory cytokine determination

The expression levels of Fe^2+^, ROS, IL-6, and TNF-α in the supernatant of MLE-12 cells and lung tissues were detected using the corresponding kits according to the manuals. MLE-12 cells were collected and centrifuged at 3000 *g* for 20 min to obtain the cell supernatant; lung tissues were minced, homogenized, lysed, and centrifuged to collect the supernatant. Iron Colorimetric Assay kit (ab83366; Abcam) and Tissue Reactive Oxygen Species Assay kit (HR8820; Balb Biotech Co., Ltd.) were used to detect the levels of Fe^2+^ and ROS in freshly resected lung tissues. Intracellular Iron Colorimetric Assay kit (E1042-100; Applygen Biotech Co., Ltd.) and Reactive Oxygen Species Assay Kit (50101ES01; Yeasen Biotech Co., Ltd.) were used to detect the Fe^2+^ and ROS levels in the MLE-12 cells. Intracellular ROS was observed under a fluorescence microscope, and the relative levels were quantified using a fluorescence microplate reader. TNF-α (ml002095-J) and IL-6 (ml063159; Mlbio Biotech Co., Ltd.) levels in both lung tissues and MLE-12 cells were detected by ELISA kits.

### Western blotting assay

RIPA buffer was used to extract proteins from cells or lung tissues. The concentration of protein was quantified using a BCA protein assay kit (GK10009; GlpBio Technology). Each lane of SDS-PAGE gel was loaded with the exact quantity of protein (40 μg) for separation and then transferred onto the PVDF membranes (Millipore). After that, the membranes were blocked by 10% skim milk and incubated with primary antibodies, such as anti-GPX4 (1:1000; abs136221; Absin), anti-NRF2 (1:1000; abs130481; Absin), anti-ACSL4 (1:1000; sc-365230; SantaCruz), anti-COX2 (1:1000; abs131986; Absin), and anti-GAPDH (1:5000; abs132004; Absin) at 4 ℃ overnight. Then, secondary goat anti-mouse IgG H&L (HRP) (ab205719; Abcam) was incubated with the membrane at room temperature for 2 h. Finally, protein bands were developed using ECL luminescence reagent (abs920; Absin) on UniCel DxI800 Automatic immune analysis system (Beckman Coulter).

### Quantitative real-time polymerase chain reaction (qRT-PCR)

The total RNA of cells and lung tissues was extracted by TRIzol® reagent (Invitrogen). qRT-PCR was carried out using Tiangen FastKing OneStep qRT-PCR Kit (KR123; Tiangen Biotech). qRT-PCR was conducted on qTOWER384G PCR System (Analytik Jena GmbH). All primers were designed and synthesized by MBL Biotech Co., Ltd. U6 was chosen as an internal reference for miRNAs. The 2^−ΔΔCt^ method was calculated to assess the RNA fold changes.

### CCK-8 assay

The cell suspension was diluted to 3 × 10^4^ cells/mL and added into each well of a 96-well plate. Next, 10 μL of CCK8 reagent (M4839; Abmole Bioscience Inc.) was added to the plate and cultured at 37 ℃ for 4 h. The optical densities were evaluated with a microplate reader at the wavelength of 450 nm.

### Luciferase reporter assay

The GPX4 luciferase reporter vectors were designed and synthesized by Hanbio Bioengineering Co., Ltd.; it included wild (WT) and mutant (MUT) types 3′-UTR region. To verify whether GPX4 could bind to miR-134-5p, a mimic/inhibitor and WT/MUT GPX4 vectors were co-transfected into the cells. After 48 h, cells were lysed to detect the luciferase activities using the Firefly & Renilla Dual Luciferase Assay Kit (F6075; Yuheng Biotech Co., Ltd.). The luciferase activity was normalized to Renilla luciferase activity.

### RNA pull-down assay

At room temperature, MLE-12 cells were collected and lysed at least 1 × 10^7^ cells, and then, probe-conjugated beads were generated by incubating the biotin-GPX4 probes with Pierce™ Streptavidin Magnetic Beads for 2 h. After that, the cell lysates were incubated with the GPX4 probes or control probes at 4 ℃ overnight. Then, rinsing with the washing buffer, magnetic beads were eluted from the complexes and detected by Western blotting. The biotinylated GPX4 probe was designed and synthesized by GenePharma.

### RNA immunoprecipitation with an anti-Ago2 antibody

GPX4 probes or control probes were transfected into the MLE-12 cells. After 24 h, the cells were lysed, and the cell lysate was collected to immune precipitate with anti-Ago2 antibody (1:1000; ab186733; Abcam) as described previously [21]. Ago2 immuno-complexes were purified with the GeneJET RNA Purification Kit (K0732; Thermo Fisher). The levels of miR-134-5p were quantified by qRT-PCR and visualized by agarose gel electrophoresis.

### Bioluminescence assay

The mice injected with FAM-labeled R8-ROS @miR-134-5p inhibitor, FAM-labeled PCC-R8-ROS @miR-134-5p inhibitor, or PBS were anaesthetized, then placed supine in the FluoView400 Fluorescence Imaging System (Guangzhou Biolight Biotechnology Co., Ltd.) to observe in vivo luminescence. After euthanasia, lung tissues were collected and imaged. The regions of interest (ROI) areas were drawn, and the luminescence intensities were shown as p/s/cm2/sr. In each group, fluorescence intensity at the ROI is expressed as the mean ± SD for five mice.

### Statistics

Each experiment was performed in triplicates. Data were analyzed by GraphPad Prism (version 9.1.1.225). The data were presented as mean ± SEM. Student’s *t*-test was performed for two groups, and analysis of variance (ANOVA) was used for multiple groups, followed by Duncan’s post hoc test. In the present study, *p* < 0.05 was deemed as a significant difference.

## Results

### Ferroptosis is involved in the hyperoxia-induced BPD mice

**Fig. 1:**
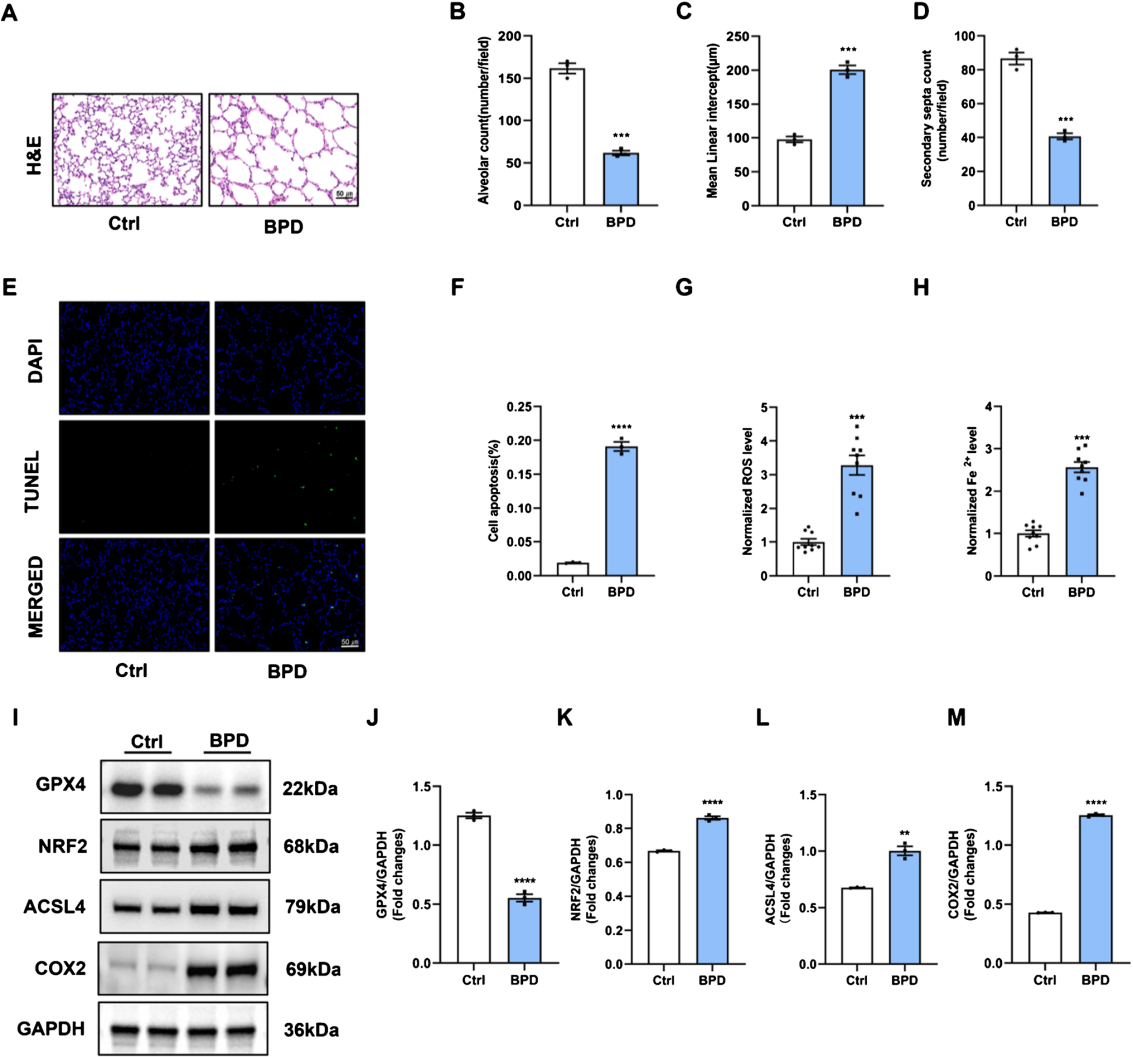
Ferroptosis is involved in the hyperoxia-induced BPD mice. **A** H&E staining assay was applied to evaluate the pathological changes of the lung tissues. Scale bar, 50 μm. **B** Quantification of the alveolar number of lung tissues. **C** Quantification of the mean linear intercept of lung tissues. **D** Quantification of the secondary septa of lung tissues. **E** TUNEL assay was used to estimate the cell apoptosis rate of the lung tissues. **F** Quantification of the cell apoptosis rate of the lung tissues. **G** ROS level and **H** Fe.^2+^ level were determined by the corresponding kits. **I** The protein levels of the ferroptosis-related genes, GPX4, NRF2, ACSL4, and COX2, were measured by Western blot assay. **J**–**M** Densitometric analysis of **J** GPX4 expression, **K** NRF2 expression, **L** ACSL4 expression, and **M** COX2 expression. Western blot densitometric values were normalized to GAPDH. The values are the mean ± SEM; *n* = 5 mice/group. The results shown were observed in at least three independent experiments. **p < 0.01, ***p < 0.001, and ***p < 0.0001

The BPD mice model was generated by exposing neonatal mice to hyperoxia (85% oxygen) for 7 days. The H&E staining images showed that the lungs from the BPD mice exhibited severe impairment of alveolar growth (Fig. 1A). In per unit area, mean linear intercept (MLI) increased, and the number of alveoli and secondary septa decreased (Fig. 1B–D). These histological changes demonstrated the successful establishment of the BPD mice model. As shown in Fig. 1E, F, cell apoptosis was significantly increased in the BPD group, indicating accelerated cell death in the BPD group. Besides, higher ROS levels and Fe^2+^ levels were also detected in the lung tissues of the BPD group (Fig. 1G, H). According to the results in Fig. 1A–H, we concluded that ferroptosis occurred in the lung tissues of mice in the BPD group. We then checked for the expression levels of ferroptosis-related genes in the lungs by Western blotting (Fig. 1I–M). The GPX4 protein expression levels were decreased significantly, along with the increased levels of NRF2, ACSL4, and COX2.

### The miR-134-5p overexpression leads to ferroptosisin vitro

**Fig. 2:**
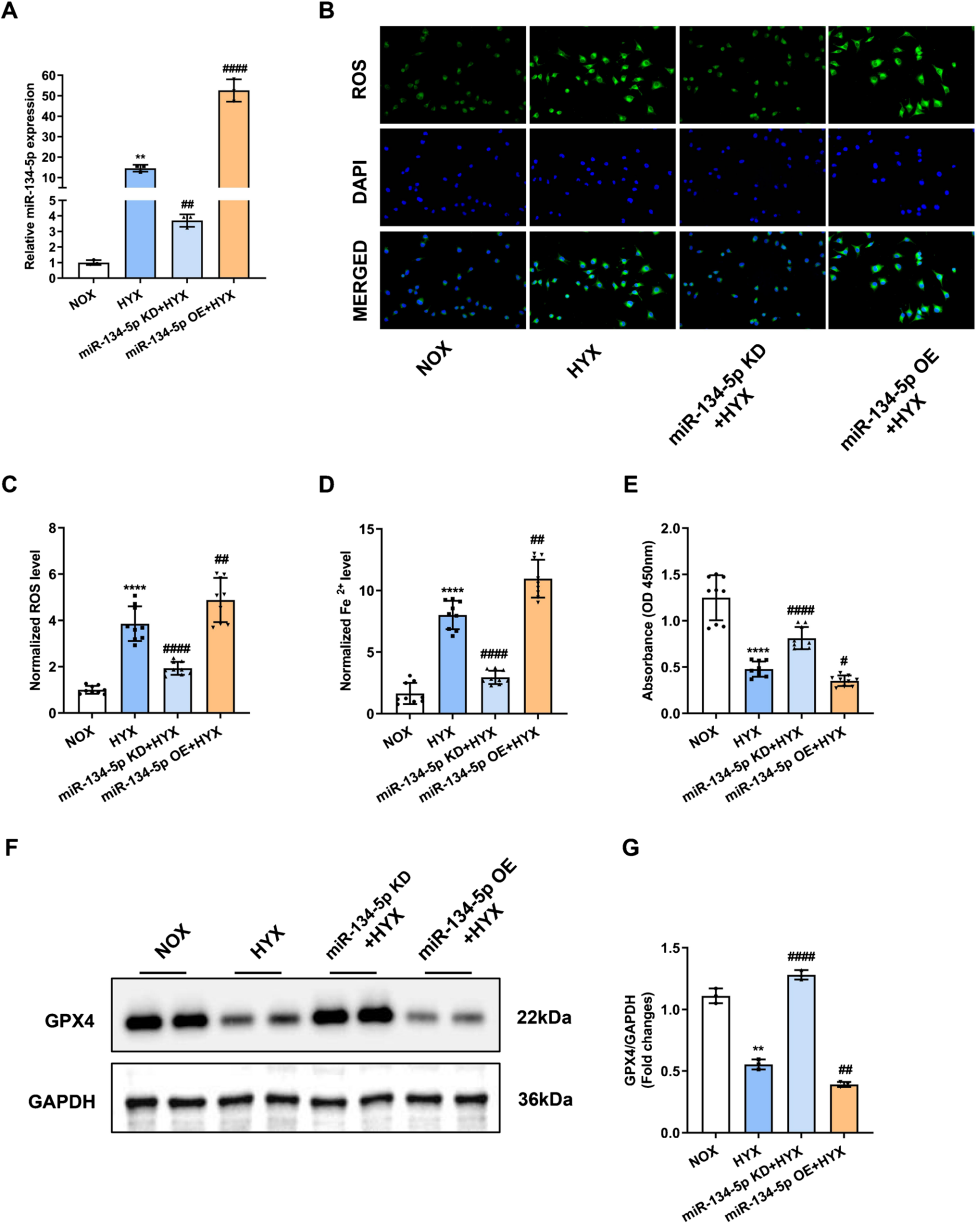
The miR-134-5p overexpression leads to ferroptosis in vitro. MLE-12 cells were transfected with miR-134-5p inhibitor (miR-134-5p knockdown, miR-134-5p KD) or miR-134-5p mimic (miR-134-5p overexpression, miR-134-5p OE) to explore the effects of miR-134-5p on ferroptosis. After 24 h of transfection, the MLE-12 cells were subjected to 24 h of hyperoxia. **A** The expression levels of miR-134-5p in MLE-12 cells were detected using qRT-PCR. **B** ROS accumulation in the MLE-12 cells was examined using the corresponding kit. The green fluorescence represented for ROS. **C** Quantification of the levels of ROS was detected using the kit. **D** Quantification of the levels of Fe^2+^ was detected using the kit. **E** Cell viabilities were determined by CCK8 assay. **F** The protein expressions of GPX4 were measured by Western blot assay. **G** Densitometric analysis of GPX4 expression. Western blot densitometric values were normalized to GAPDH. The experiment was set with 3 independent replicates. **p < 0.01, ****p < 0.0001 vs. NOX; #*p* < 0.05, ##*p* < 0.01, ####*p* < 0.0001 vs. HYX

We consulted a previous BPD-related miRNA profiling study [16], where miR-134-5p was upregulated in the BPD mice, and it was predicted to target GPX4 using miRWalk website (mirwalk.umm.uni-heidelberg.de). To investigate the relationship between miR-134-5p and ferroptosis in BPD, MLE-12 cells were transfected with miR-134-5p inhibitor (miR-134-5p knockdown, miR-134-5p KD) or miR-134-5p mimic (miR-134-5p overexpression, miR-134-5p OE) to explore the effects of miR-134-5p on ferroptosis. After 24 h of transfection, the MLE12 cells were subjected to 24 h of hyperoxia. The miR-134-5p expression levels were quantified in each group using qRT-PCR (Fig. 2A). Compared to the normoxia group (NOX), miR-134-5p level in the hyperoxia group (HYX)was significantly elevated. Moreover, the miR-134-5p OE group had the most apparent increase. We could also detect the accumulation of ROS in MLE-12 cells. As shown in Fig. 2B, the hyperoxia group exhibited higher intensity of green fluorescence which was consistent with the results in vivo; miR-134-5p inhibitor reduced ROS level, while miR-134-5p mimic promoted ROS level. The intracellular levels of ROS in the four groups were quantified in Fig. 2C. Further, the levels of miR-134-5p represented a similar trend to the ROS level. Fe^2+^ levels were increased in the miR-134-5p overexpression group and the hyperoxia group. However, enforced expression of miR-134-5p promoted Fe^2+^ accumulation in hyperoxia (Fig. 2D). Reverse results were observed in the cell viability of MLE-12 cells (Fig. 2E). Furthermore, as shown in Fig. 2F, G, GPX4 expression was prominently inhibited in the MLE-12 cells from the hyperoxia group; miR-134-5p inhibitor abolished the effect of hyperoxia on GPX4, while miR-134-5p exhibited a synergistic effect with hyperoxia. This result indicated that the overexpression of miR-134-5p led to GPX4 depletion in cells. Since the levels of ROS, Fe^2+^, and GPX4 are biomarkers of ferroptosis, we concluded that the overexpression of miR-134- 5p initiated the ferroptosis of MLE-12 cells.

### MiR-134-5p targeted for GPX4

**Fig. 3:**
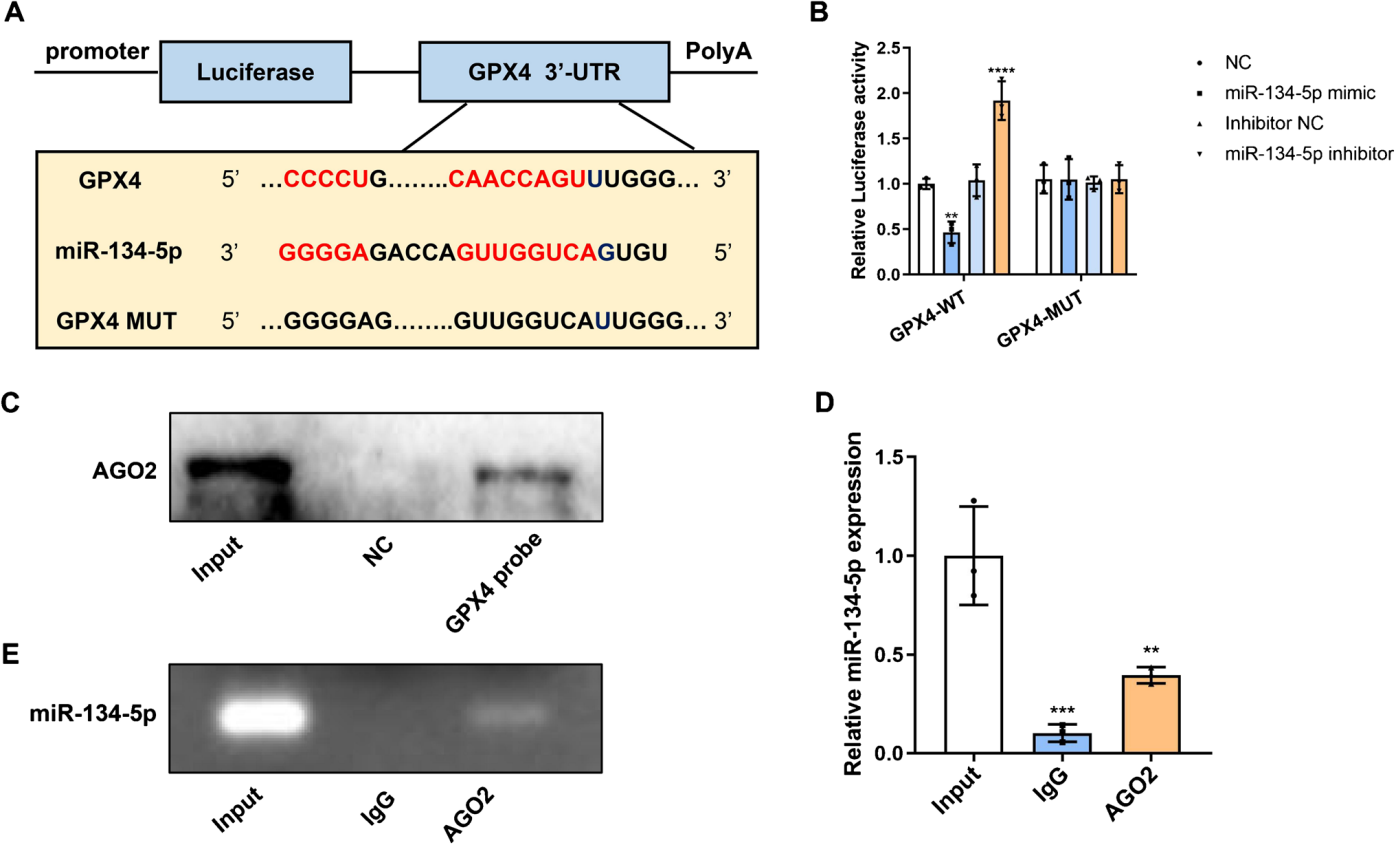
MiR-134-5p targeted for GPX4. **A** The binding sequences of miR-134-5p and GPX4 were listed. The binding sites of wild type GPX4 (GPX4-WT) were mutated and cloned into the luciferase reporter vectors as mutant type GPX4 (GPX4-MUT). **B** The luciferase activities of MLE-12 cells were determined after transfected with miR-134-5p mimic/inhibitor and GPX4-WT/MUT. **C** The expression of Ago2 was measured by Western blot after pull-down assay. **D** The expression level of miR-134-5p was examined by qRT-PCR and **E** agarose gel electrophoresis after immunoprecipitation. Three experiments were performed for each assay. **p < 0.01, ***p < 0.001, ****p < 0.0001

The predicted binding site of miR-134-5p to GPX4 is shown in Fig. 3A. The sequences CAACCAGUU and CCCCU were mutated and cloned into the luciferase vector (GPX4-MUT) to exam the binding effect. miR-134-5p mimic notably decreased the luciferase activity of GPX4-WT, but miR-134-5p inhibitor promoted the luciferase activity. However, in the GPX4-MUT group, either miR-134-5p inhibitor or mimic showed no significant impact on luciferase activity (Fig. 3B). These results confirmed the binding relationship between GPX4 and miR-134-5p. We know that Ago2 is essential for miRNAs recruiting to the cytoplasmic RNA-induced silencing complex (RISC). Ago2 facilitates miRNA binding to the target site on mRNA, cleaving the miRNA-mRNA duplex [22]. The pull-down assay results demonstrated that GPX4 probes could recruit Ago2 proteins (Fig. 3C), suggesting the potential of GPX4 to form the miRNA-mRNA duplex. miR-134-5p level in the immunoprecipitates was significantly increased (Fig. 3D, E), which also confirmed the miR-134-5p-GPX4 duplex.

### Assembly and identification of PCC-R8-ROS@miR-134-5p inhibitor

**Scheme 1:**
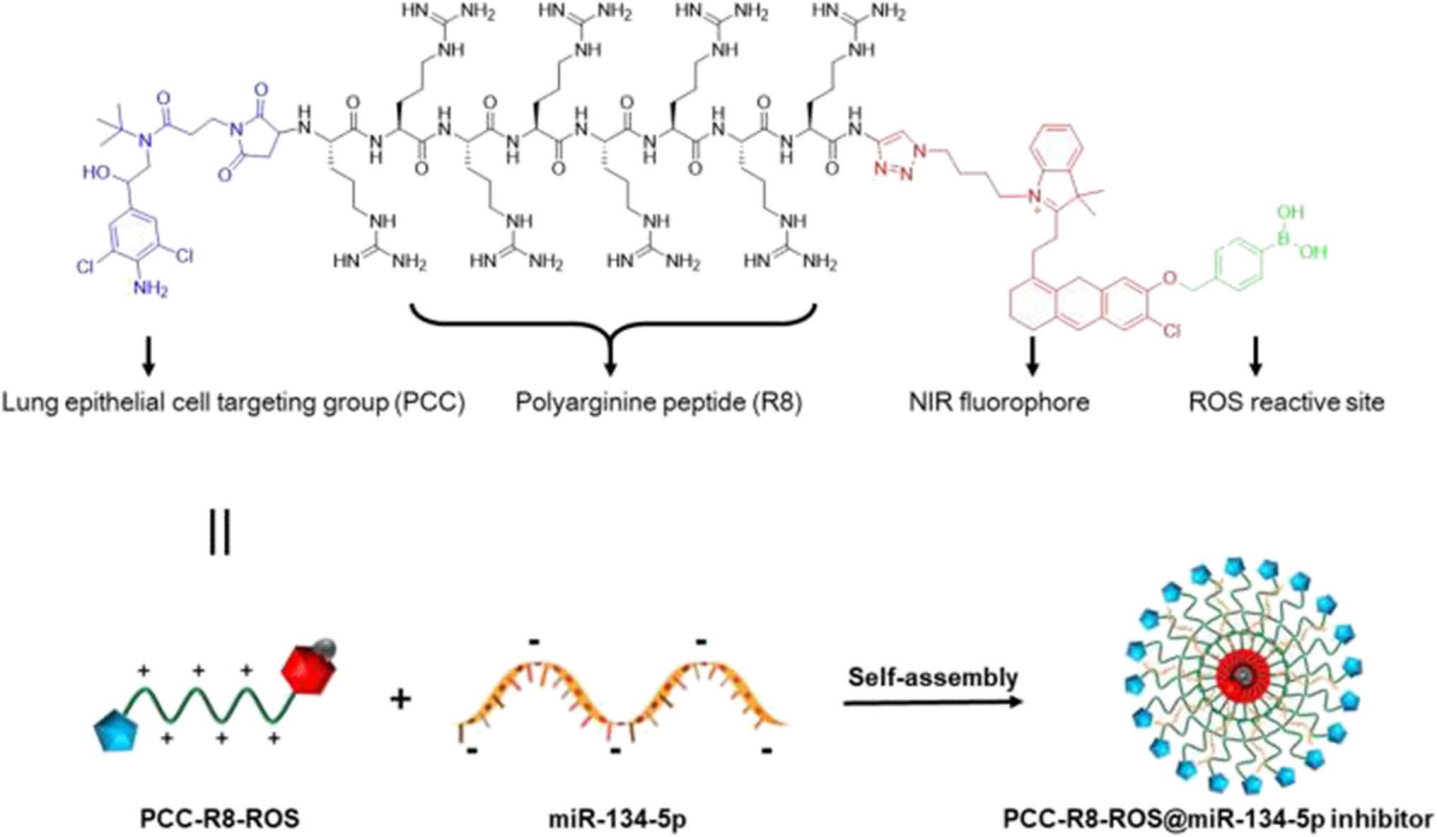
The assembly process diagram of pulmonary epithelial cell-targeting self-assembly nanocarrier

**Fig. 4:**
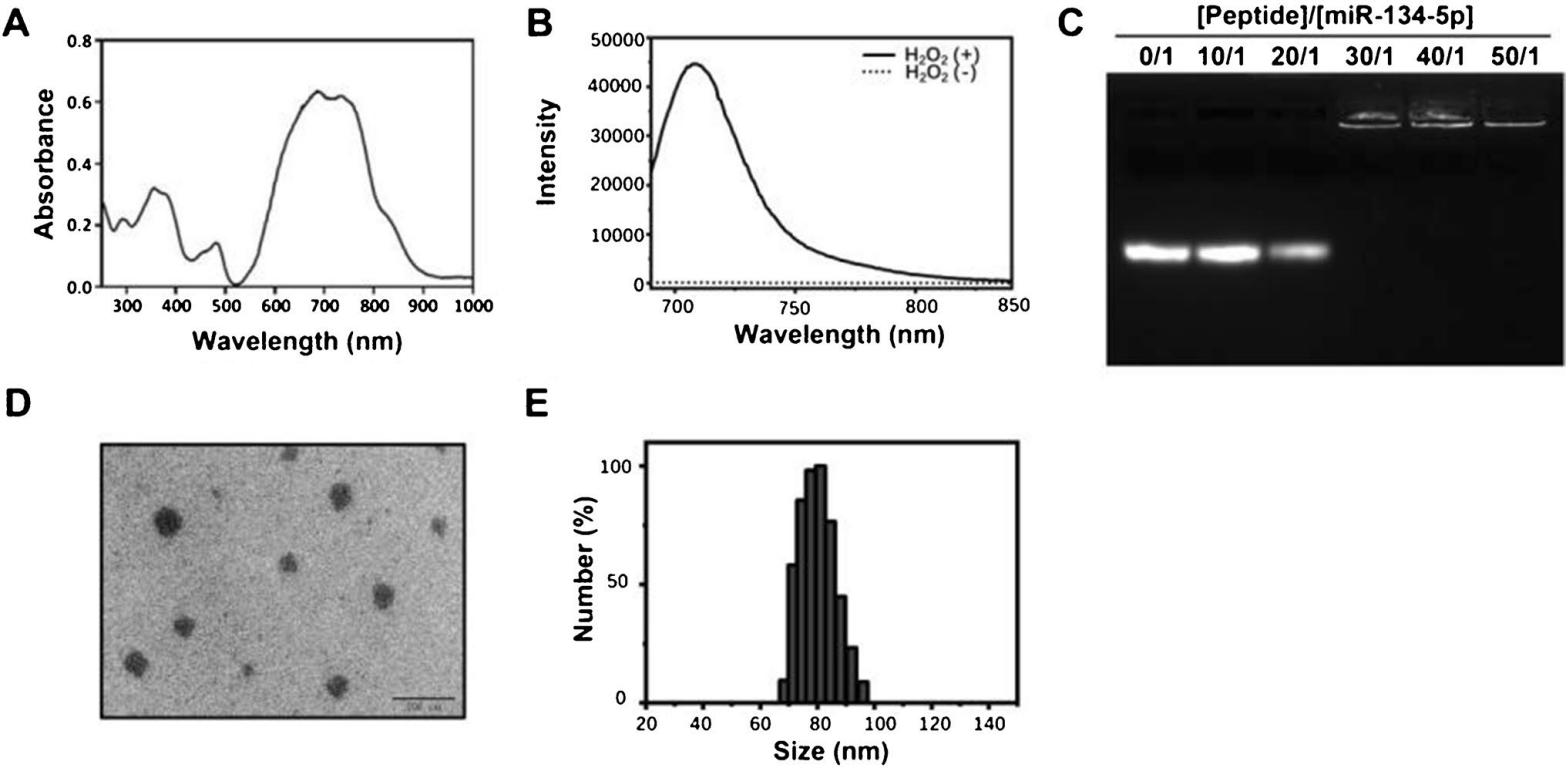
Assembly and identification of PCC-R8-ROS@miR-134-5p inhibitor. **A** UV–vis absorption of PCC-R8-ROS. **B** The fluorescence spectra of PCC-R8-ROS before and after incubation with H2O2. **C** The migration of miR-134-5p on the agarose gel. **D** Size of PCC-R8-ROS@miR-134-5p inhibitor was measured by transmission electron microscopy (TEM) and **E** dynamic light scattering (DLS)

Inspired by the effect that miR-134-5p induced ferroptosis through inhibiting GPX4, we designed a nanocarrier (PCC-R8-ROS, Scheme 1) to deliver miR-134-5p inhibitor for BPD theranostic applications efficiently. The nanocarrier contained three key components: (1) a covalently attached clenbuterol for pulmonary epithelial cell targeting (PCC); (2) a polyarginine peptide (R8) to bind miR-134-5p inhibitor and enhance cell permeability; and (3) a ROS responsive pro-fluorophore for NIR imaging (Scheme 1). The ROS-responsive pro-fluorophore was conjugated to the short peptide via Cu(I)-catalyzed azide-alkyne cycloaddition (CuAAC). The PCC-R8-ROS and its intermediates were identified by matrix-assisted laser desorption/ionization mass spectrometry (MALDI-MS) (Figure S1A). The UV–Vis-NIR spectra of PCC-R8-ROS showed the characteristic absorption peak corresponding to that of NIR pro-fluorophore (Fig. 4A). The ROS responsiveness was tested by mixing H_2_O_2_ with the nanocarrier. After H_2_O_2_ activation, a significant NIR fluorescence enhancement was observed (Fig. 4B), mirroring the high reactivity to ROS.

The nanocarrier (PCC-R8-ROS) was mixed with different concentrations of miR-134-5p inhibitors to form the assembly, followed by a gel electrophoresis assay to quantify the best-assembled ratio. As shown in Fig. 4C, the migration of miR-134-5p on the gel gradually decreased with the increase in the ratio of nanocarrier to miR-134-5p, from 0:1 to 50:1. The binding at a ratio of 30:1 completely blocked the migration of miR-134-5p, and this binding ratio was hence fixed for all subsequent experiments. The nanocomplex was precipitated by centrifugation; it further confirmed that the supernatant contained negligible free peptide conjugate and free miRNA. Before incubating with cells, the readily formed nanocomplex were used without an extra purification step. The size of the PCC-R8-ROS@miR-134-5p inhibitor was further measured using dynamic light scattering (DLS) and TEM (Fig. 4D, E). Thus, the ease of preparation of this nanocomplex makes it an ideal carrier for miRNA delivery intracellularly.

### PCC-R8-ROS-miR-134-5p inhibitor efficiently delivered miR-134-5p inhibitors to MLE-12 cells to suppress ferroptosis

**Fig. 5:**
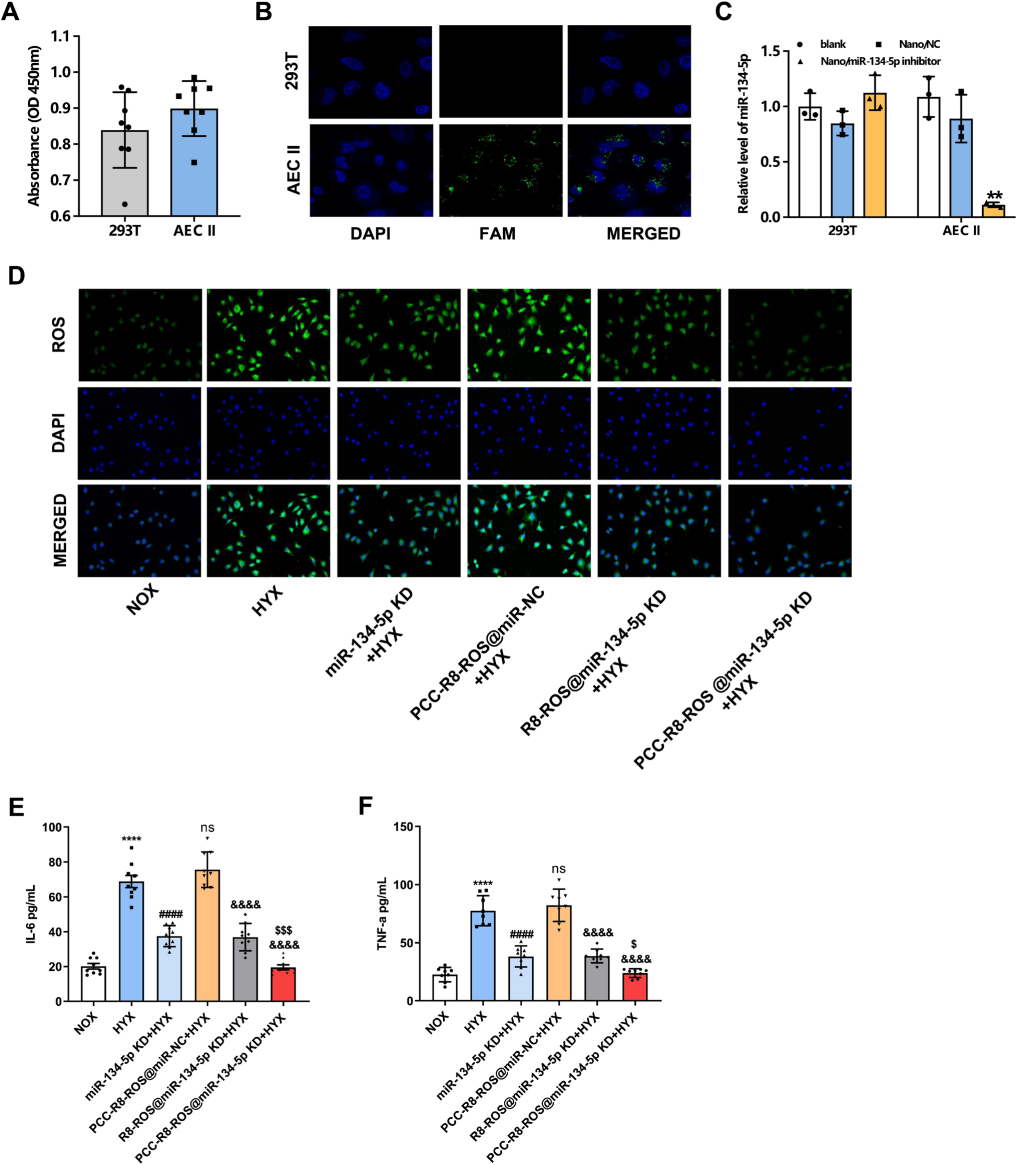
PCC-R8-ROS-miR-134-5p inhibitor efficiently delivered miR-134-5p inhibitors to MLE-12 cells to suppress ferroptosis. **A** 293T cells and MLE-12 cells were incubated with PCC-R8-ROS-miR-134-5p inhibitor for 24 h. After that, cell viabilities were determined using CCK8 assay. **B** The carboxyl fluorescein (FAM)-labeled PCC-R8-ROS-miR-134-5p inhibitors were observed in 293T cells and MLE-12 cells under a fluorescence microscope. **C** The expression levels of miR-134-5p in blank control 293T cells and MLE-12 cells, or cells transfected with PCC-R8-ROS@NC or PCC-R8-ROS@miR-134-5p inhibitor. **p < 0.01 vs. PCC-R8-ROS@NC. **D** The ROS accumulation was also observed in MLE-12 cells transfected with miR-134-5p inhibitor, PCC-R8-ROS @NC, R8-ROS@miR-134-5p inhibitor, or PCC-R8-ROS@miR-134-5p inhibitor. **E**, **F** The secretions of IL-6 and TNF-α were detected by ELISA kits. The experiment was set with 3 independent replicates. ****p < 0.0001 vs. NOX; ^####^*p* < 0.0001, ^ns^*p* > 0.05 vs. HYX; ^&&&&^*p* < 0.0001 vs. PCC-R8-ROS @NC + HYX, ^$^*p* < 0.05, ^$$$^*p* < 0.001 vs. R8-ROS@miR-134-5p KD + HYX

We next performed in vitro experiments to evaluate the efficiency of the PCC-R8-ROS @miR-134-5p inhibitor. Cytotoxicity of PCC-R8-ROS@miR-134-5p inhibitor was tested on both 293T cells and MLE-12 cells using CCK8 assay. No significance was observed between the OD values of 293T cells and MLE-12 cells, which implied PCC-R8-ROS @miR-134-5p inhibitor was non-cytotoxic (Fig. 5A). FAM-labeled PCC-R8-ROS @miR-134-5p inhibitor was incubated to verify the targeting effect with 293T and MLE-12 cells. As shown in Fig. 5B, green puncta were observed in MLE-12 cells, while negligible fluorescence was found in 293T cells, suggesting that PCC-R8-ROS @miR-134-5p inhibitor could successfully target pulmonary epithelial cells. The efficacy of PCC-R8-ROS-miR-134-5p inhibitor was further demonstrated by silencing miR-134-5p. Furthermore, PCC-R8-ROS@miR-134-5p inhibitor-treated MLE-12 cells significantly decreased in the levels of miR-134-5p compared to the PCC-R8-ROS@NC group, indicating the efficient intracellular delivery of miR-134-5p inhibitors (Fig. 5C). However, the miR-134-5p levels in 293T cells were not influenced by PCC-R8-ROS@miR-134-5p inhibitor or PCC-R8-ROS@miR-NC. Together, the PCC-R8-ROS@miR-134-5p inhibitor efficiently targeted lung epithelial cells with high intracellular delivery efficacy. ROS levels in the MLE-12 cells were subsequently quantified. miR-134-5p inhibitor reduced the ROS level compared to the hyperoxia group (HYX). Both R8-ROS @miR-342-5p inhibitor and PCC-R8-ROS @miR-342-5p inhibitor reduced ROS levels in the MLE-12 cells compared to the PCC-R8-ROS @miR-NC group (Fig. 5D), which partly verified our hypothesis that silencing miR-134-5p protected cells from ferroptosis. Surprisingly, cells treated with PCC-R8-ROS@miR-134-5p inhibitor exhibited even weaker fluorescence than the R8-ROS @miR-342-5p inhibitor group, indicating ROS elimination’s high efficiency (Fig. 5D). The same trend was observed in the expressions of IL-6 and TNF-α (Fig. 5E, F), confirming the dual role of miR-134-5p inhibition in eliminating ROS and anti-inflammation. Together, the PCC-R8-ROS@miR-134-5p inhibitor exhibited more effective functions in blocking the ferroptosis pathway by eliminating ROS with reduced inflammation.

### PCC-R8-ROS@miR-134-5p inhibitor efficiently targeted for lung regions

**Fig. 6:**
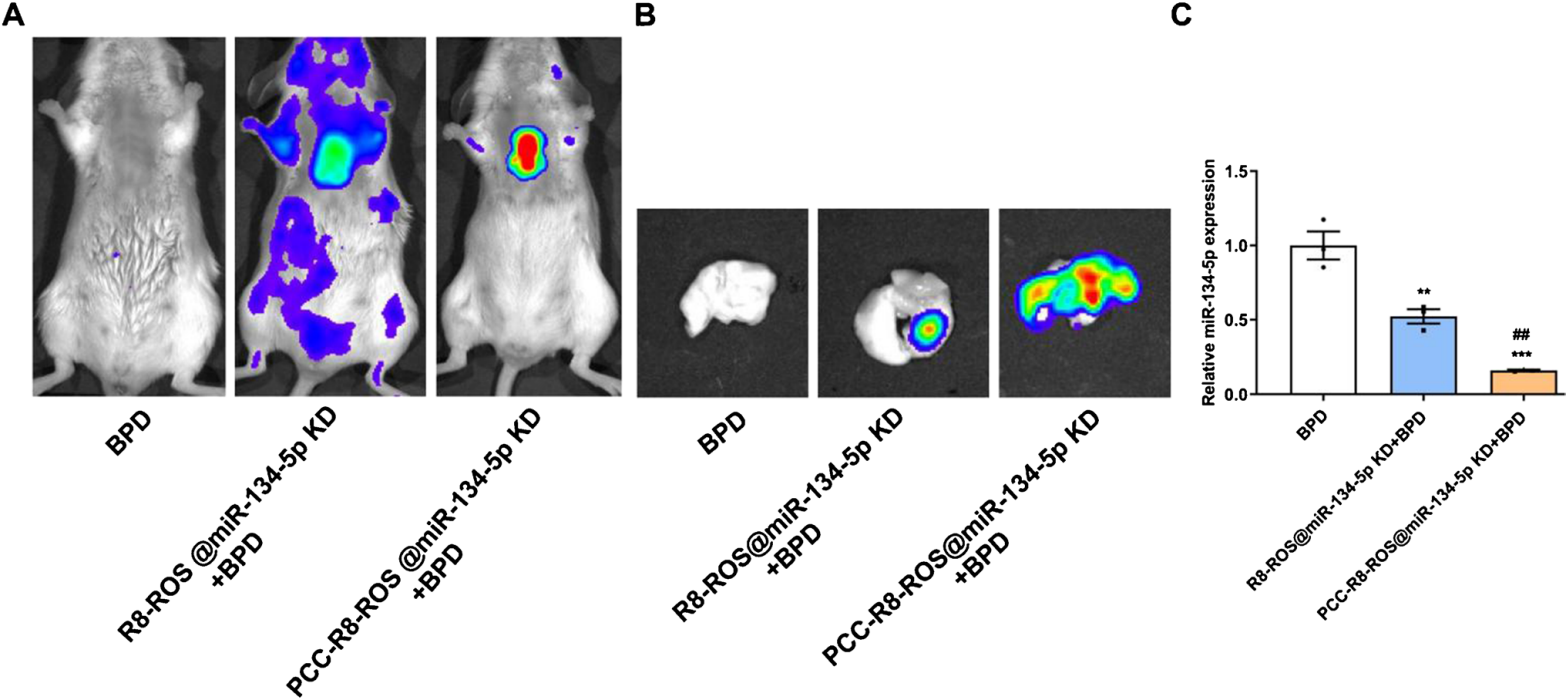
PCC-R8-ROS@miR-134-5p inhibitor efficiently targeted for lung regions. **A** Bioluminescence assay was performed to locate R8-ROS@miR-134-5p inhibitor and PCC-R8-ROS@miR-134-5p inhibitor in vivo, and representative images were displayed. **B** The lung tissues were resected and subjected to bioluminescence assay, and representative images were displayed. **C** The expression levels of miR-134-5p were determined in the lung tissues. **p < 0.01, ***p < 0.001 vs. BPD; ^##^*p* < 0.01 vs. R8-ROS@miR-134-5p KD + BPD

Encouraged by the above cell experiments, we investigated the targeting ability of nanocarriers in vivo. PCC-R8-ROS@miR-134-5p inhibitor and R8-ROS@miR-134-5p inhibitor were injected intravenously into the BPD mice, and their distribution was monitored by fluorescence imaging. The mice have sacrificed afterwards for ex vivo imaging. Localized fluorescence was detected in the lungs of the PCC-R8-ROS @miR-134-5p inhibitor group, while the images from the R8-ROS@miR-134-5p inhibitor group exhibited widely distributed fluorescence throughout the body (Fig. 6A). Moreover, the PCC-R8-ROS@miR-134-5p inhibitor showed intense fluorescence in the lung tissue, while the R8-ROS@miR-134-5p inhibitor only partially fluoresced in the lung tissue (Fig. 6B). Besides, PCC-R8-ROS@miR-134-5p inhibitor markedly downregulated miR-134-5p in the lung tissues (Fig. 6C), reflecting the effective targeted miRNA inhibitor delivery for theranostic applications.

### PCC-R8-ROS@miR-134-5p inhibitor alleviated BPD in vivo

**Fig. 7:**
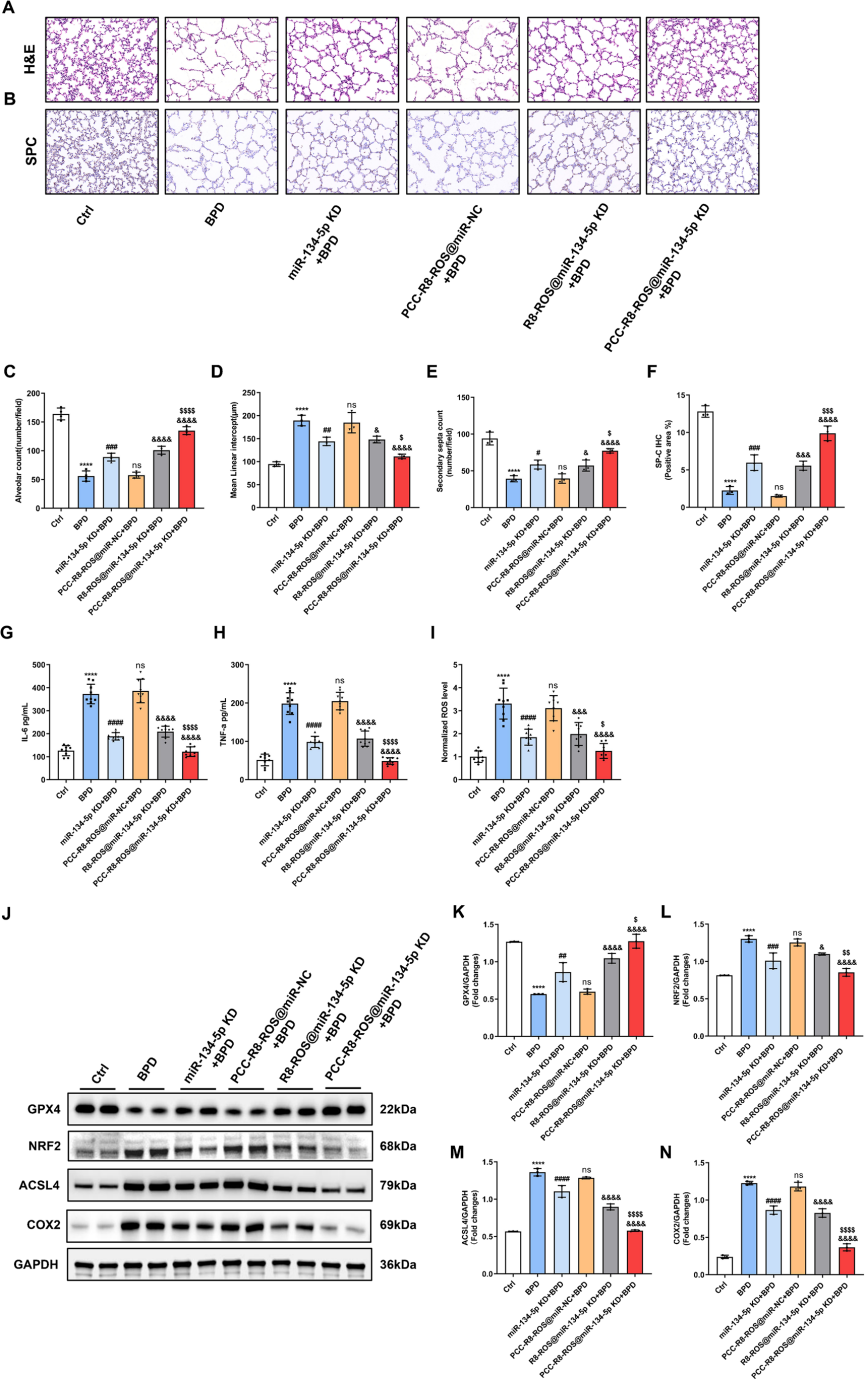
PCC-R8-ROS@miR-134-5p inhibitor alleviated BPD in vivo. **A** H&E staining assay was carried out to evaluate the histological damage on lung tissues. Scale bar, 50 μm. **B** The deposition of the type II alveolar epithelial cell marker, SP-C, was evaluated by immunohistochemistry. **C** Quantification of the alveolar number of lung tissues. **D** Quantification of the mean linear intercept of lung tissues. **E** Quantification of the secondary septa of lung tissues. **F** Quantification of the SP-C immunohistochemistry. **G**, **H** The levels of inflammatory cytokines, IL-6 and TNF-α, were determined by ELISA kits. **I** The levels of ROS were detected using the corresponding kit. **J** The protein expressions of ferroptosis-related genes were detected by Western blot. **K**–**N** Densitometric analysis of **K** GPX4 expression, **L** NRF2 expression, **M** ACSL4 expression, and **N** COX2 expression. Western blot densitometric values were normalized to GAPDH. The values are the mean ± SEM; the experiment was set with 3 independent replicates. ****p < 0.0001 vs. Ctrl, ^#^*p* < 0.05, ^##^*p* < 0.01, ^###^*p* < 0.001, ^####^*p* < 0.0001, ^ns^*p* > 0.05 vs. BPD; ^&^*p* < 0.05, ^&&&^*p* < 0.001, ^&&&&^*p* < 0.0001 vs. PCC-R8-ROS @NC + BPD; ^$^*p* < 0.05, ^$$^*p* < 0.01, ^$$$$^*p* < 0.0001 vs. R8-ROS@miR-134-5p KD + BPD. The experiment was set with 3 independent replicates

The therapeutic effect of the PCC-R8-ROS@miR-134-5p inhibitor was evaluated in a BPD mouse model. The H&E staining revealed that all the miR-134-5p inhibitor treatments ameliorated the damage on lung histomorphology. Notably, the PCC-R8-ROS@miR-134-5p inhibitor-treated group was most effective in ameliorating lung morphometry (Fig. 7A, C, D, and E), highlighting the high efficiency of the targeted nanocarrier. Moreover, images of SP-C immunohistochemistry staining in Lung tissue sections also demonstrated that the PCC-R8-ROS @miR-134-5p inhibitor complex could efficiently prevent the type II alveolar epithelial cells from being damaged (Fig. 7B, F). As displayed in Fig. 7G–I, a significant decreased levels in IL-6, TNF-α, and ROS were detected in the PCC-R8-ROS@miR-134-5p inhibitor group, reflecting alleviated inflammation in the lung. PCC-R8-ROS@miR-134-5p inhibitor almost restored the ferroptosis-related protein expressions in the BPD model to the normal level (control group) (Fig. 7J–N). Notably, the PCC-R8-ROS@miR-134-5p inhibitor complex showed negligible toxicity to normal tissues (Figure S1D), which further supported the promising potential of developing new therapeutical strategies based on this self-assembled nanocarrier of therapeutic miRNAs. Hence, the PCC-R8-ROS@miR-134-5p inhibitor complex showed a promising application in treating BPD with reduced off-target effects.

## Discussion

The incomplete development of the antioxidant system in premature infants makes them more susceptible to oxidative stress damage, leading to ferroptosis, a form of cell death caused by Fe^2+^-dependent lipid peroxidation. During BPD, alveolar epithelial cells are the primary affected cells, so inhibiting ferroptosis in alveolar epithelial cells is the most effective strategy to improve the disease outcomes. In addition to GSH/GPX4-mediated ferroptosis regulatory mechanisms, Nrf2 regulatory networks were reported to control both iron homeostasis and lipid peroxidation [23]. The balance between antioxidant and oxidative pools directly determines the severity of ferroptosis and subsequent alveolar epithelial cell regeneration. In present study, we verified that the excessive miR-134-5p induced the decreased protein level of GPX4 in BPD mice. Further, we synthesized a self-assembly nanocarrier system of miR-134-5p inhibitor that targeted the intracellular ROS generated in the lung epithelial cells.

The critical proteins involved in scavenging ROS, such as GSH, GPX4, and SOD, are reduced in the peripheral blood and bronchoalveolar lavage fluid of premature infants due to the incomplete development of their antioxidant system [24]. Some premature infants with respiratory insufficiency need oxygen support, and in infants with a history of postnatal asphyxia, the increase of free iron results in the production of excessive ROS [25]. Hence, improving the activity of the antioxidant system in premature infants is the core challenge in preventing and treating BPD. In this study, we demonstrated that the protein expression of GPX4 was significantly decreased in experimental BPD models. As we know, inhibition of GPX4 leads to the oxidation of several polyunsaturated fatty acids (PUFAs) and the production of fatty acid-free radicals, which results in cellular ferroptosis [26]. Previous studies have elucidated that GPX4 is downregulated in many lung diseases, such as acute lung injury [27], radiation-induced lung fibrosis [28], and non-small cell cancer [29].

Moreover, ACSL4 is involved in the biosynthesis and remodeling of PUFA-PE in the cell membrane; the increased expression of ACSL4 indicated aggravated ferroptosis [30]. The miR-134-5p was previously predicted to be upregulated in the BPD mice, and this was validated in the current study for the first time. Besides, we found that inhibition of miR-134-5p markedly suppressed the development of ferroptosis in hyperoxia-induced MLE-12 cells, while overexpression of miR-134-5p was able to rescue this trend. The regulation between GPX4 and miR-134-5p was subsequently demonstrated. miRNAs have been shown to play vital roles in the progression of BPD by influencing the expressions of target genes. For example, miR-574-3p was significantly inhibited in BPD infants, which resulted in the overexpression of ADM in BPD [30]. The levels of miR-421 were significantly elevated in BPD mice, which exacerbated inflammatory responses and promoted cell apoptosis in the lung tissues by inhibiting the expression of Fgf10 [31]. In addition, after BPD modeling in the mice, the levels of miR-342-5p increased; however, the expression of Spred3 was inhibited due to the targeting relationship [13]. A lot of the research on miR-134-5p is focused on oncology. For instance, miR-134-5p was downregulated in osteosarcoma, and the target gene MBTD1 was overexpressed, promoting cancer cell viability and inhibiting cell apoptosis [32]. In contrast, miR-134-5p accelerated lung adenocarcinoma metastasis via targeting DAB2 [33]. In our study, we identified that miR-134-5p promoted BPD via facilitating ferroptosis; it sheds a new insight into the functioning of miR-134-5p.

Since transfection of miR-134-5p inhibitor significantly contributed to the amelioration of ferroptosis in BPD, the efficiency of transporting miR-134-5p inhibitor to MLE-12 cells was a significant concern. In the current research, we designed and synthesized a nanocarrier of miR-134-5p inhibitors that could efficiently target lung epithelial cells (Scheme 1). miR-134-5p inhibitors were packaged into the nanocarriers through peptide self-assembly. Peptide self-assembly refers to the molecular recognition between peptide molecules or between certain fragments in the peptide molecular structure under specific external stimuli through multiple non-covalent intermolecular bonds.

During the self-assembly process, the synergistic effect of driving forces such as hydrogen bonding, electrostatic interactions, π-π stacking, hydrophobic forces, non-specific van der Waals forces, and chiral dipole–dipole interactions [4] enables precise control over the molecular structure of peptides. In our research, we proved that when the nanocarriers were assembled with miR-134-5p inhibitors at the ratio of 30:1, the migration of miR-134-5p was inhibited. The ability of these nanocarriers to selectively target lung epithelial cells was attributed to the clenbuterol, and ROS-reactive fluorophore was conjugated to locate the carriers. Cytotoxicity and the efficient delivery into the cell cytoplasm through endosomal escape are the two significant challenges associated with self-assembly nanocarriers. Herein, we proved that the PCC-R8-ROS @miR-134-5p inhibitors showed no significant cytotoxicity on MLE-12 cells compared to 293T cells. PCC-R8-ROS@miR-134-5p inhibitors were observably accumulated in MLE-12 cells. Further, MLE-12 cells incubated with PCC-R8-ROS@miR-134-5p inhibitors exhibited notably lower levels of miR-134-5p.

Furthermore, the images from the bioluminescence assay proved that carriers conjugated with PCC were preferentially enriched in the lungs of mice, demonstrating the specific targeting ability of PCC-R8-ROS@miR-134-5p inhibitors, which resulted in the increased transporting efficiency of miR-134-5p into the cytoplasm. The self-assembly nanocarriers are being used to transport miRNAs to treat intractable diseases, such as myocardial infarction [34], colorectal cancer [35], and hepatocellular cancer [36]. Our findings laid a foundation for the potential application of the nanocarriers in treating BPD.

Although this miRNA delivery system improves the tissue distribution and site-specific localization of miRNA, it also faces challenges. The main challenges of nanocarriers are related to its low encapsulation efficiency because the peptide/miR-134-5p ratios should be higher than 30/1 for complete encapsulation of miR-34a in its complex with the peptide conjugate PCC-R8-ROS. This may be because miRNAs have a high affinity for water, they diffuse quickly into the aqueous phase when using nanoprecipitation are used, resulting in low encapsulation efficiency. As such, increasing the electropositivity of peptide conjugates may facilitate the improvement of the encapsulation efficiency.

## Conclusions

In the current study, we revealed that ferroptosis was one of the pathogeneses of BPD, and upregulation of miR-134-5p in BPD was responsible for the decreased expression of GPX4. In order to ameliorate BPD effectively, we have developed a self-assembled and pulmonary epithelial cell-targeting PCC-R8-ROS nano-carrier to realize targeted delivery of miR-134-5p, real-time imaging of intracellular function of the delivered miR-134-5p, as well as BPD therapy. The PCC-R8-ROS nano-carriers showed high delivery efficiency of miR-134-5p inhibitors, which suggested the potential for further development as a therapeutic approach for treating BPD. The convenient construction and successful applications of this new type of miRNA nano-complex may provide the opportunity to explore various bio-medical applications of synthetic miRNA mimics. However, in the future, the major challenges in improving encapsulation efficiency are also needed to be addressed for efficient miRNA delivery.

### Supplementary Information

Below is the link to the electronic supplementary material.

Supplementary file1 (DOCX 3964 KB)

## Data Availability

The authors confirm that the data supporting the findings of this study are available within the article [and/or its supplementary materials].
