# Supplementary material for: Self-assembled miR-134-5p inhibitor nanoparticles ameliorate experimental bronchopulmonary dysplasia (BPD) via suppressing ferroptosis
Source: Mikrochim Acta. 2023 Nov 30;190(12):491. doi: 10.1007/s00604-023-06069-3 (PMC10687138; doi:10.1007/s00604-023-06069-3)
Supplement: Supplementary file 1 — Supplementary file1 (DOCX 3964 KB) [file 604_2023_6069_MOESM1_ESM.docx]

# Electronic Supplementary Material

**Self-assembled miR-134-5p inhibitor nanoparticles ameliorate experimental bronchopulmonary dysplasia (BPD) via suppressing ferroptosis**

Jiang Lan^1,4 #^, Xu Chen^2, #^, Fengdan Xu^3#^, Fangfei Tao^2^, Liyuan Liu,^1^ Rui Cheng^2, *^,

Ning Li^3^*, Ya Pan^1,*^

*1* *Shenzhen Longhua Maternity and Child Health Care Hospital, Shenzhen 518000, China.*

*2 Children's Hospital Affiliated to Nanjing Medical University (Nanjing Children's Hospital), Nanjing 210008, China.*

*3 Dongguan Children’s Hospital Affiliated to Guangdong Medical University, Dongguan 523325, China*

*4 Hongqiao International Institute of Medicine, Tongren Hospital, Shanghai Jiao Tong University School of Medicine, Shanghai 200336, China*

**^#^** These authors contributed equally to this work.

*** Correspondence:** panya1979@163.com, [chengrui350@163.com](mailto:chengrui350@163.com,), mean163@163.com.


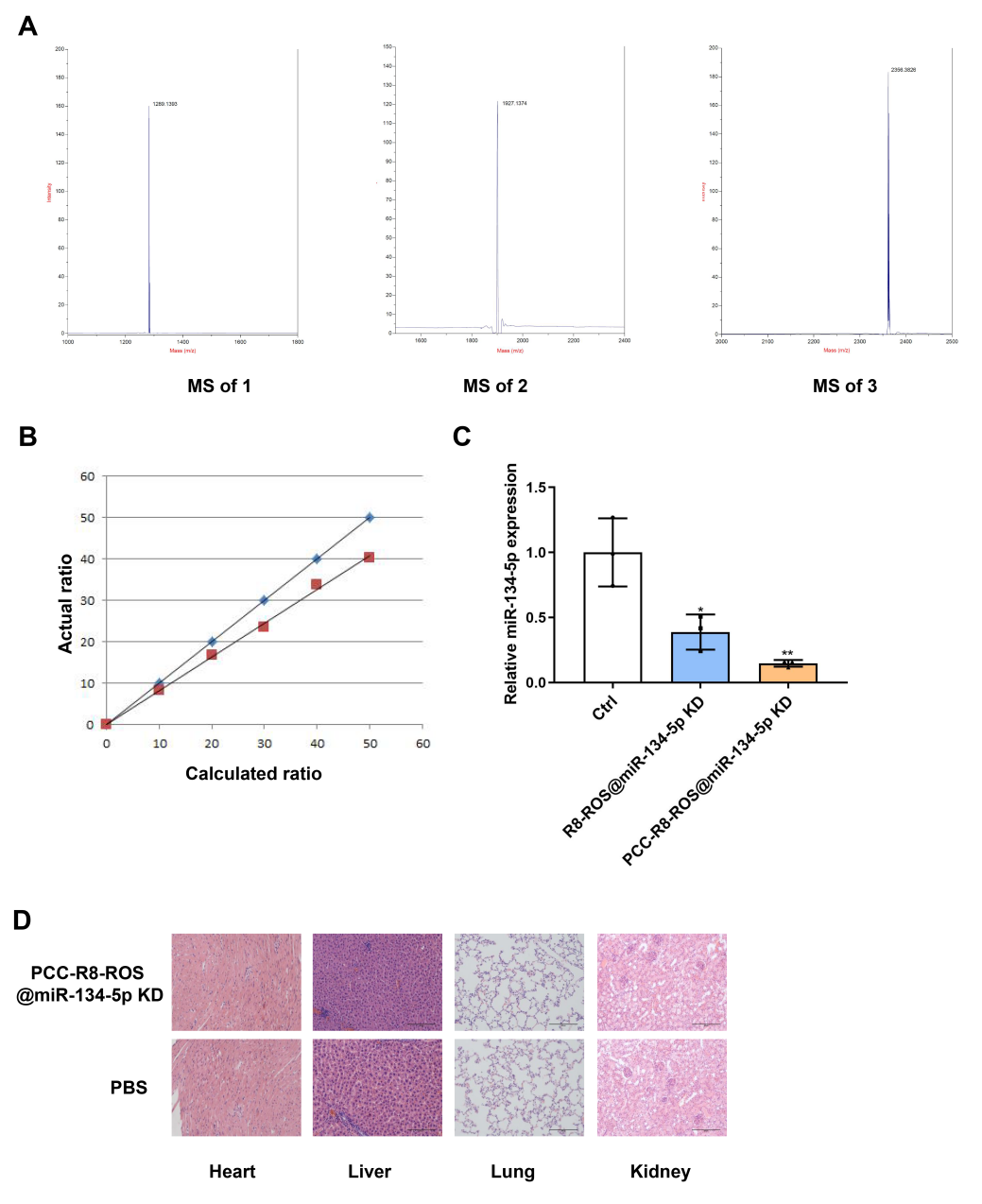


Figure S1. (A) The PCC-R8-ROS and its intermediates were characterized by matrix-assisted laser desorption/ionization mass spectrometry (MALDI-MS). (B) Actual molar ratios in nanocomplexes formed by self-assembly of miR-134-5p with different amounts of polypeptide probes. (C) The expression levels of miR-134-5p were determined in the MLE-12 cells. (D) It is noteworthy that the PCC-R8-ROS@miR-134-5p inhibitor complex showed negligible toxicity to normal tissues according to ex vivo examination on different tissues.

**
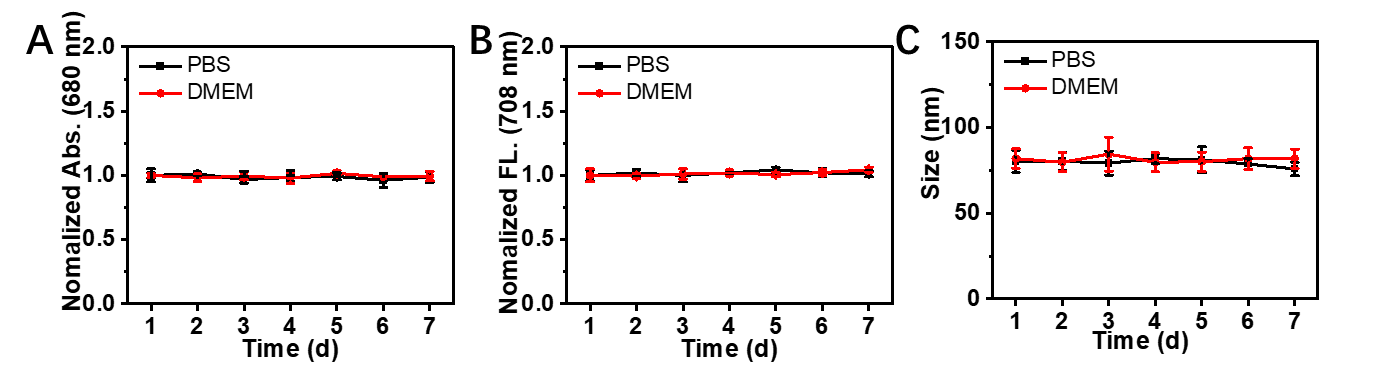
**

Figure S2. Stability evaluation of the nanoparticles. (A-B) Monitoring of absorbance (680 nm), fluorescence (708 nm) and (C) hydrodynamic diameter of nanoparticles following incubation in PBS buffer or DMEM cell culture medium at r.t. for 7 days.

Figure S3**.** The expression levels of miR-134-5p were determined in the lung tissues of mice treated with nanoparticles for different time (0, 24, 48, 72 h and 96 h).
